# Core – shell upconversion nanoparticle – semiconductor heterostructures for photodynamic therapy

**DOI:** 10.1038/srep08252

**Published:** 2015-02-05

**Authors:** Qing Qing Dou, Adith Rengaramchandran, Subramanian Tamil Selvan, Ramasamy Paulmurugan, Yong Zhang

**Affiliations:** 1 Institute of Materials Research and Engineering (IMRE), A * STAR (Agency for Science, Technology and Research), 3 Research Link, Singapore 117602; 2 Molecular Imaging Program at Stanford, Bio-X Program, Stanford University School of Medicine, Palo Alto, California 94304, USA; 3 Department of Biomedical Engineering, Faculty of Engineering, National University of Singapore, 9 Engineering Drive 1, Singapore 117575

## Abstract

Core-shell nanoparticles (CSNPs) with diverse chemical compositions have been attracting greater attention in recent years. However, it has been a challenge to develop CSNPs with different crystal structures due to the lattice mismatch of the nanocrystals. Here we report a rational design of core-shell heterostructure consisting of NaYF_4_:Yb,Tm upconversion nanoparticle (UCN) as the core and ZnO semiconductor as the shell for potential application in photodynamic therapy (PDT). The core-shell architecture (confirmed by TEM and STEM) enables for improving the loading efficiency of photosensitizer (ZnO) as the semiconductor is directly coated on the UCN core. Importantly, UCN acts as a transducer to sensitize ZnO and trigger the generation of cytotoxic reactive oxygen species (ROS) to induce cancer cell death. We also present a firefly luciferase (FLuc) reporter gene based molecular biosensor (ARE-FLuc) to measure the antioxidant signaling response activated in cells during the release of ROS in response to the exposure of CSNPs under 980 nm NIR light. The breast cancer cells (MDA-MB-231 and 4T1) exposed to CSNPs showed significant release of ROS as measured by aminophenyl fluorescein (APF) and ARE-FLuc luciferase assays, and ~45% cancer cell death as measured by MTT assay, when illuminated with 980 nm NIR light.

Core-shell nanoparticles (CSNPs) have been attracting greater attention in recent years for biomedical applications^1^,^2^,^3^, owing to their diverse chemical compositions in achieving novel multi-functionalities. Photodynamic therapy (PDT) is emerging as a new treatment option for cancer therapy. PDT utilizes photosensitizers whose electrons can be excited to high energy level upon light illumination^4^,^5^. These excited electrons sensitize the ambient oxygen molecules to reactive oxygen species (ROS), which can destroy protein, lipid and nucleic acid to induce cell death. There are three major limitations in traditional PDT which uses UV-visible light for illumination: (i) a limited treatment depth due to shallow penetration in tissue of the UV-visible light, (ii) nonspecific tissue damage by natural light exposure to photosensitizer, and (iii) photo-damage of normal tissues by harmful UV light.

Up-conversion nanoparticles (UCNs) have been used in PDT, and showed promising applications over the traditional PDT^6^,^7^,^8^,^9^. Due to the unique electronic configuration of the doped lanthanide ions, near infrared (NIR) light (976 ~ 980 nm) is used for the illumination of UCNs. Recently, an enhanced upconversion luminescence in Tm^3+^-doped NaYF_4_ UCNs was achieved by high excitation irradiance and high activator concentration^10^. After absorbing longer wavelength NIR light, UCNs emit short wavelength UV or visible light. By designing UCNs as a transducer for sensitizing the attached photosensitizer, the aforementioned limitations of traditional UV PDT could be circumvented, improving the penetration depth of NIR light to tissues without photo-damage.

Currently, there are four major methods used to combine UCNs with photosensitizers for PDT. The first method is based on encapsulating photosensitizers into mesoporous silica coated on UCNs^6^,^7^. Besides, covalent conjugation^8^,^11^, electrostatic interactions^9^ and lipid micelles encapsulations^12^ have also been used to anchor the photosensitizers to the UCNs for PDT. These methods involve several time-consuming steps, starting from synthesis to surface modification, and photosensitizer loading or attachment. In the case of mesoporous silica coating method, the major limitation is the aggregation of the synthesized NPs after calcinations in obtaining mesoporous silica structure, which hinders the circulation of the NPs in serum and blood. In other methods, such as physical adsorption and electrostatic attraction, the premature release of photosensitizers might occur before the NPs reach to the desired target site.

ZnO NPs have been reported as efficient photosensitizers^13^,^14^,^15^ since ZnO can sensitize the ambient oxygen to produce ROS. Coincidently, bandgap of ZnO (3.3 eV)^16^,^17^ matches perfectly with the emission peaks (330 ~ 370 nm) of one the most efficient UCNs (composed of NaYF_4_:Yb,Tm). Here we report a new approach to the synthesis of NaYF_4_:Yb,Tm (UCN)@ZnO in a core-shell manner for PDT applications. Compared to other nanomaterials used for PDT, our UCN@ZnO CSNPs exhibit four major advantages. Firstly, when compared to the conventional UV-visible light sensitized PDT, the PDT with CSNPs could reach deeper tissues since 980 nm NIR light for triggering PDT falls perfectly in the biological optical window. Secondly, light source (UCN) and photosensitizer (ZnO) can be easily incorporated in the core-shell structure. Thirdly, photosensitizer-loading efficiency can be controlled by CSNPs synthesis, as the photosensitizer is directly coated on the UCN core. Finally, the core-shell architecture eases photosensitizer loading procedures without considering much about the hydrophilicity, carrying charge and functional groups. Therefore, this design largely reduces the fabrication cycle and greatly improves the photosensitizer loading efficiency. In this work, we have used citrate ligand exchange method to obtain water soluble UCN@ZnO CSNPs in order to evaluate the therapeutic effect by measuring the ROS generation under NIR light irradiation.

We have also constructed genetically encoded FLuc reporter gene- expressed under antioxidant response element (ARE) containing ARE promoters derived from NAD(P)H (nicotinamide adenine dinucleotide phosphate) quinone oxidoreductase 1 (NQO1) and glutathione S-transferase (GST) genes, which are capable of measuring the activated antioxidant response signaling in cells by monitoring ROS production. In general, the induction of ROS in cells by any means can activate cellular antioxidant responsive genes, such as NAD(P)H (nicotinamide adenine dinucleotide phosphate) quinone oxidoreductase 1 (Nqo1), Glutamate-cysteine ligase, catalytic (Gclc), Heme oxygenase-1 (HO-1), glutathione S-transferase (GST), DP-glucuronosyltransferase (UGT), and Multidrug resistance-associated proteins (Mrps), especially to ameliorate cells from adverse effects^18^,^19^,^20^. Hence, the activation of the above genes in cancer cells can be indirectly used for measuring the cells response to PDT. In this work, we have measured the release of ROS in MDA-MB-231 breast cancer cells after treating them with CSNPs and excitation with NIR light by ARE-Luciferase reporter system. The Renilla luciferase reporter gene expression under an ubiquitin promoter showing no response to antioxidant signaling was used to normalize the ARE signaling measured by firefly luciferase.

## Results

### Design of UCN@ZnO CSNPs

**Figure 1:**
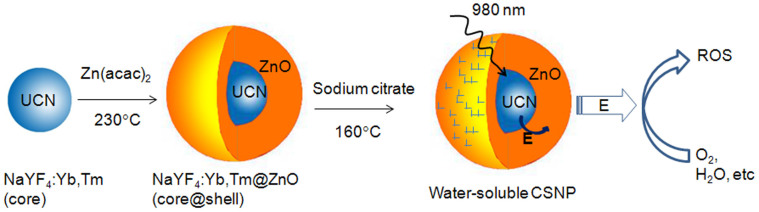
Schemetic showing the synthesis. Hydrophilic UCN@ZnO core-shell nanoparticles (CSNPs) and their therapeutic route in producing the reactive oxygen species (ROS) upon the excitation at 980 nm NIR light. E refers to energy transfer.

Fig. 1 depicts the design and synthetic strategy of UCN@ZnO CSNPs. To obtain CSNPs, UCNs were synthesized first and used as the core. Next, Zn(acac)_2_ was used as Zn precursor for the crystalline ZnO shell growth on the UCN core. As ZnO and UCN are fabricated in a core-shell manner, ZnO can utilize the UV-light emitted from the UCN after being excited by NIR light (980 nm), and sensitize ROS generation from oxygen for PDT. As the NIR light penetration is much larger than UV-Vis light in biological tissues, ROS generated in cells containing CSNPs upon NIR irradiation can induce cell death. Therefore, the specificity of PDT will be largely enhanced without much adverse side effects. Meanwhile, the other emission peaks of NaYF_4_:Yb,Tm UCN (not matching with the wavelength of ZnO absorbance) can be used simultaneously for bioimaging.

### Structural characterization of CSNPs

**Figure 2:**
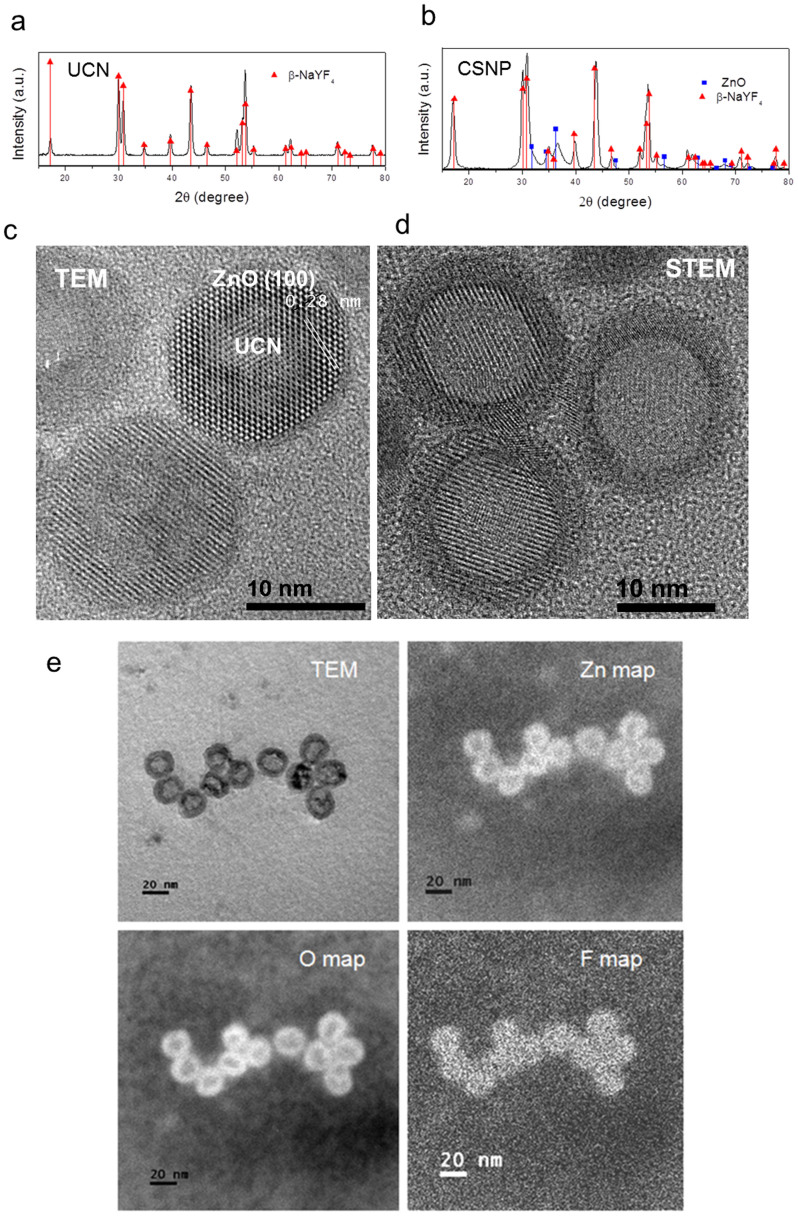
Structural characterization. XRD patterns of (a) core UCN before coating and (b) UCN@ZnO CSNPs. (c) High-resolution TEM and (d) STEM images of CSNPs. (e) TEM and elemental mapping of CSNPs by STEM. The brightness of the image represents the concentration of the element.

The size distribution of as-synthesized UCN@ZnO CSNPs (after purification) was measured by a nano-zeta sizer based on dynamic light scattering (DLS) principle. The polydispersity index (PDI) of the synthesized CSNPs was in the range of 0.139 to 0.201, and was consistent with different batches of samples (Supplementary Figs. S1, S2). This result suggested that the prepared CSNPs were uniform in size and monodispersed in cyclohexane, which primarily confirmed the homogeneity of the CSNPs. Figs. 2a, 2b show the XRD patterns of UCN core and CSNPs, respectively. The XRD pattern of CSNPs (Fig. 2b) can be assigned to two crystalline hexagonal phases of NaYF_4_ (the host lattice of UCN core) and ZnO (shell).

From the above two analyses, it was still early to exclude the possibility of forming two different types of NPs in similar size and further draw a conclusion that the core-shell structure was formed. To gain more insights, a high-resolution transmission electron microscope (HRTEM) was used as a direct proof to confirm the core-shell structure of the as-synthesized NPs. From the HRTEM image (Fig. 2c), the inter-planar distance on the periphery of core UCN was measured to be 0.28 nm, corresponding to the (100) crystal plane of hexagonal phase ZnO^21^,^22^. This observation confirms that ZnO is coated on the core UCN, yielding CSNPs. Additionally, the energy dispersive X-ray analysis (EDX, Supplementary Fig. S3) carried out on the sample in Figure 1b showed the presence of Zn, O and Y elements in the CSNPs. To obtain a higher contrast TEM image, the NPs were also examined by scanning transmission electron microscope (STEM), whose contrast is directly related to the atomic number of the element. In the STEM image (Fig. 2d), it can be clearly seen that a continuous shell with uniform thickness of around 2–3 nm is coated on the UCN core. The STEM elemental mapping (Fig. 2e) shows that both Zn and O elements are located only on the periphery of the CSNPs, while fluorine is present in the core. All these observations from HRTEM and STEM confirm that a 2–3 nm ZnO shell is coated successfully on the UCN core.

### Luminescent properties of CSNPs

**Figure 3:**
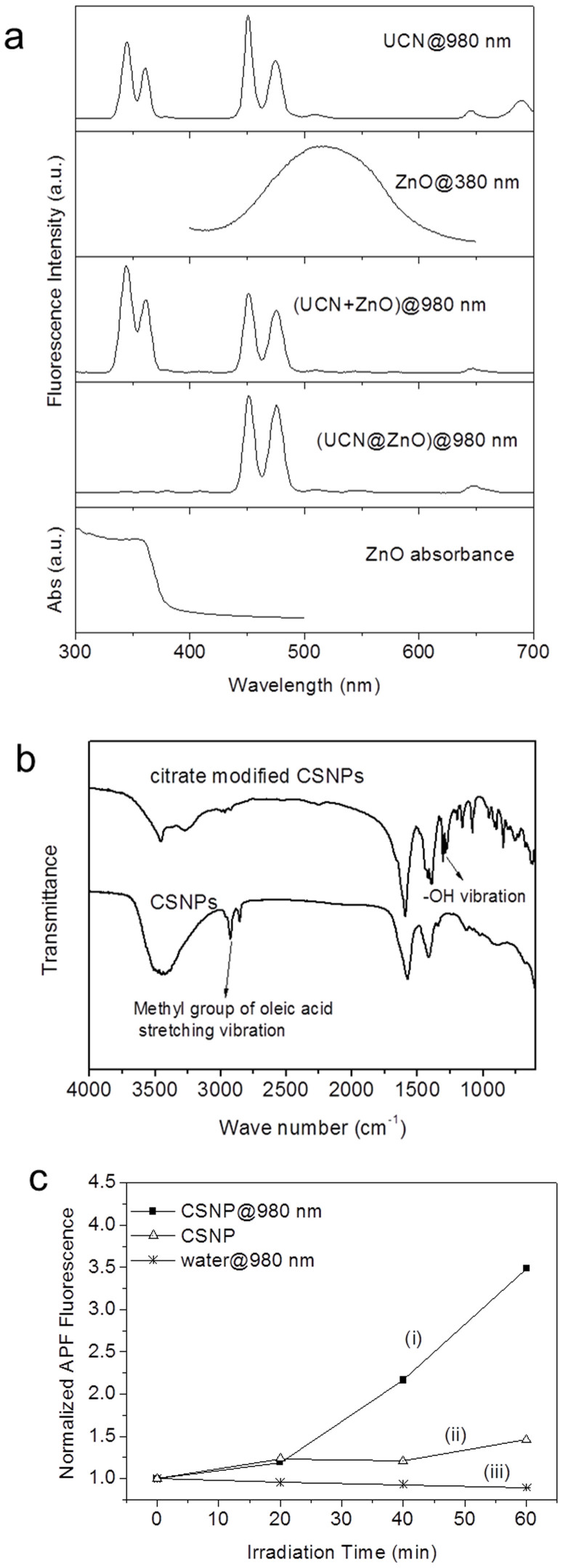
Optical, FTIR and ROS generation. (a) Upper panel: Emission spectra of core UCN, UCN@ZnO CSNPs, UCN + ZnO physical mixture, excited by 980 nm laser with the power density of 17.7 W/cm^2^, and ZnO NPs at 380 nm excitation. Bottom panel: UV-vis absorbance spectrum of ZnO NPs. (b) FTIR spectra of as-synthesized (hydrophobic) and citrate modified (hydrophilic) water-soluble CSNPs. (c) Normalized APF fluorescence (corresponding to the reactive oxygen species, ROS production) versus laser irradiation time: (i). Citrate modified CSNPs under portable diode CW 980 nm laser with the power density of 2.16 W/cm^2^. (ii). Citrate modified CSNPs without irradiation. (iii). Control sample (water) with irradiation.

The emission spectra of CSNPs and core UCN are compared in Fig. 3a. It can be seen that the emission peaks of UCN at 330–370 nm are dramatically suppressed in CSNPs. Coincidently, this wavelength range matches perfectly with the absorbance wavelength of ZnO. Conversely, the emission spectrum derived from a simple physical mixing of individual NPs of UCN and ZnO is the reminiscent of the emission of pure UCN. This indicates that there is no energy transfer between UCN and ZnO when they are physically mixed in solution. This also confirms that the core-shell structure is formed in as-synthesized NPs because the energy transfer occurs between NPs that are in close proximity^23^.

### Hydrophilic Conversion of CSNPs

As the CSNPs were synthesized in organic solvent, the hydrophobic surface had to be modified with hydrophilic ligands in order to perform the PDT. The ligand exchange with anhydrous sodium citrate was employed for producing water-soluble CSNPs. The success of the hydrophilic conversion of the CSNPs was monitored by the Fourier transform infrared spectroscopy (FTIR) by comparing the chemical bonds on the CSNPs surface before and after the conversion (Fig. 3b). The peak at 3000 cm^−1^, corresponding to the vibration stretching of methyl group^24^ in oleic acid, disappeared after the surface modification with citrate. Meanwhile, new peaks at around 1250 cm^−1^ corresponding to the hydroxyl group from citrate^25^,^26^ appeared, confirming the successful ligand exchange with citrate. Unlike the surface modification with silica coating, the citrate-modified CSNP solution in water was stable and remained clear and transparent.

### PDT evaluation of CSNPs in solution

The PDT application potential of the hydrophilic CSNPs was evaluated by measuring the ROS induced by 980 nm irradiation. The amount of ROS was measured with a light-induced oxidation resistant dye, 3′-(p-aminophenyl) fluorescein (APF). This dye remains non-fluorescent until it reacts with ROS such as hydroxyl radical, peroxynitrite anion, and hypochlorite anion^27^,^28^,^29^. Thus, the ROS amount is directly proportional to the fluorescence intensity of APF dye. First, CSNPs were dispersed in water. After adding APF into the CSNPs solution, it was excited with 980 nm irradiation for 1 h, and the fluorescence of APF was recorded at every 20 min interval. As shown in Fig. 3c, the APF fluorescence intensity of the citrate-modified CSNPs increased with the time of NIR light exposure, indicating the increased generation of ROS with time. In contrast, citrate modified CSNPs without NIR irradiation, and APF in water solution did not show much increase in APF fluorescence. These results confirm that the UCN@ZnO CSNPs can sensitize ROS generation efficiently with 980 nm irradiation, indicating the PDT application potential of the CSNPs with NIR light.

### Cytotoxicity of CSNPs

**Figure 4:**
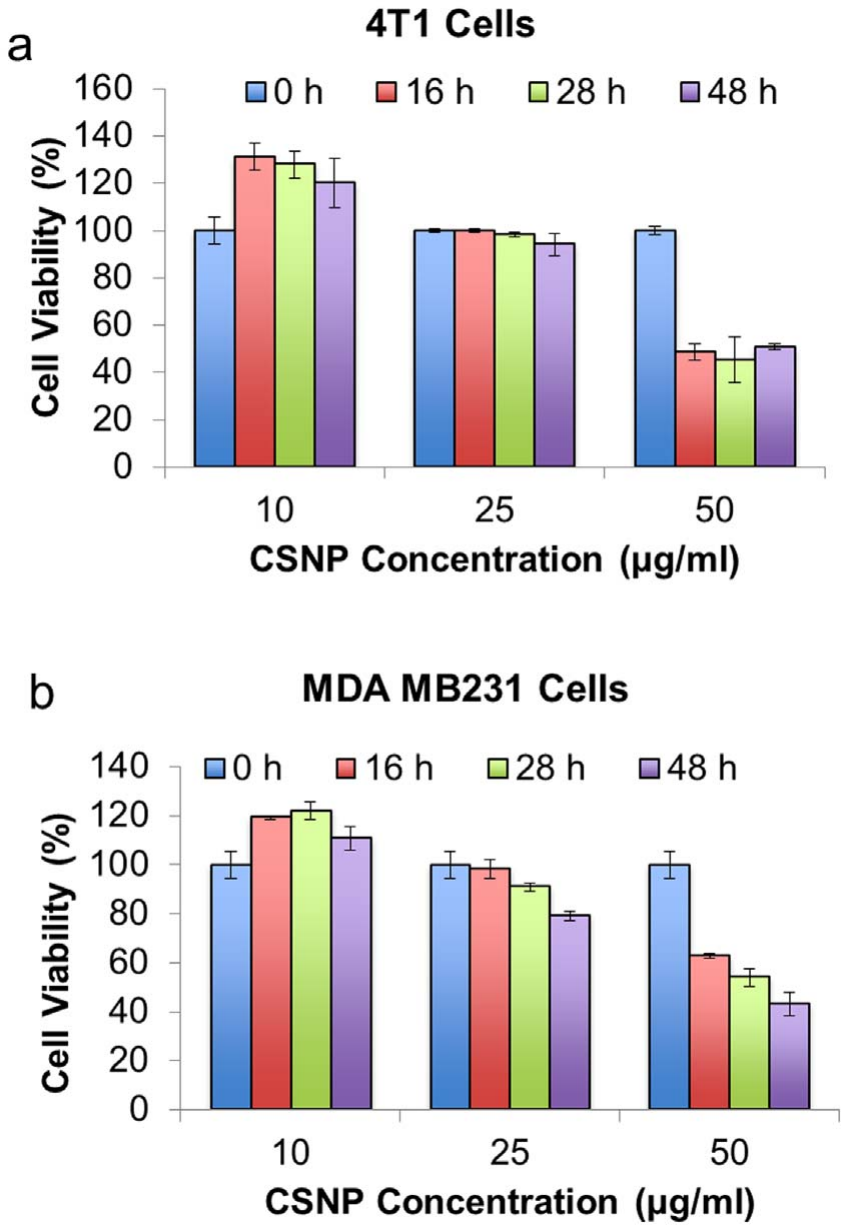
Cytotoxicity evaluation of CSNPs. Cell viability of (a) 4T1 and (b) MDA-MB-231 breast cancer cells incubated with different concentration of CSNPs (10, 25 and 50 μg/mL) at different incubation times (16, 26 and 48 h) without 980 nm NIR light exposure.

The toxicity of the CSNPs without NIR irridiation to cells was evaluated on both 4T1 and MDA-MB-231 cell lines. After incubating with 10, 25 and 50 μg/mL CSNPs, the viability of cells was recorded at three different time points (16, 28 and 48 h). The results showed that there was no significant impact on cell growth when the cells were incubated with low concentration (10 μg/mL) of CSNPs. Both the cell lines showed great compatibility up to 25 μg/mL CSNPs (Figs. 4a, 4b), but MDA-MB-231 cells showed rising toxicity when incubated with CSNPs for longer time (28 h in Fig. 4b). Both cell lines showed signficant reduction in cell viablity when cells were incubated with 50 μg/mL CSNPs. Both cell lines exhibited no cell death with 10 μg/mL CSNPs even after longer incubation time, indicating a safe concentration range of CSNPs that can be used for PDT with 980 nm NIR light irradiation. Hence, the particle concentration of 10 μg/mL was chosen for further light activations studies in this work.

### Basal ROS level measurement by ARE-Luciferase assay

**Figure 5:**
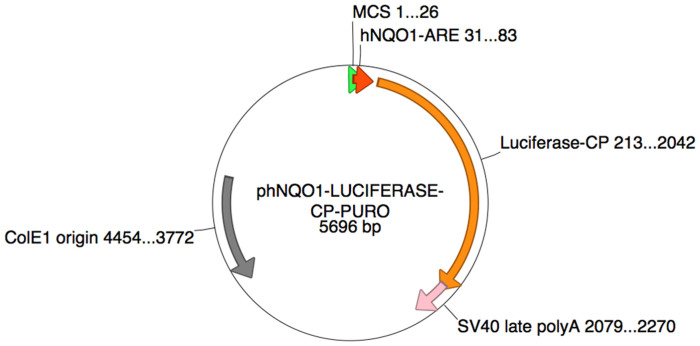
Schematic drawing of ARE-luciferase vector construct. MCS, multicloning site; hNQO1-ARE, ARE promoter derived from hNQO1 gene; Luciferase, FLuc reporter gene; SV40 late ployA, Poly Adenylation signal sequence from simian virus 40 late gene; ColE1, Prokaryotic origin of replication sequence from bacterial Colicin E1 plasmid. The numbers appears next to each gene sequence is the nucleotide position of respective element.

In general, there will be a basal level of ROS produced in normal cells at all times due to aerobic respiration^30^,^31^. This can affect the detection limit of ROS in CSNPs treated cells after activation by NIR irradiation. Hence, it is important to evaluate the basal level of ROS released in cells in order to assess the ROS induced by CSNPs. ARE-Luciferase reporter gene assay^32^ was used to measure the basal level of ROS in live cells. A schematic of ARE-luciferase vector construct used for this study is shown in Fig. 5. As shown, the vector construct possesses ARE-promoter driving the expression of FLuc reporter gene. The release of ROS in cells activates antioxidant response genes through ARE-promoter, including the ARE-FLuc reporter vector trasnsfected in these cells. The quantity of activated luciferase signal measured in cells was directly proportional to the amount of ROS produced and the expression of various endogenous antioxidant response genes. The antioxidant response genes are a group of genes, which are activated by ROS and electrophile, released by the cells in response to xenobiotic chemical exposure. NQO1 and GST1 genes derived luciferase reporter gene expression was used to measure ROS basal levels in cells before CSNPs treatment.

The 4T1 (Supplementary Figs. S4a, S4b) and MDA-MB-231 (Figs. S4c, S4d) cells transfected with NQO1- or GST-Luciferase constructs were treated with four different concentrations of CSNPs (0, 5, 10 and 25 μg/mL). Luciferase activity was measured to indirectly monitor the ARE-signaling induced by the basal ROS at 12, 24 and 48 h incubation with CSNPs. The normalized NQO1-luciferase activity at constant CSNPs concentration at different time points showed significant difference, especially at later time points (24–48 h) (Supplementary Figs. S4a, S4c). This indicated that the ARE-luciferase signal derived from NQO1 promoter is not suitable for measuring ROS release during the PDT treatment of CSNPs. In contrast, the ARE derived from GST1- promoter showed consistently low background signal even at high concentration of CSNPs (25 μg/mL) used for this study, in both 4T1 and MDA-MB-231 cells (Supplementary Figs. S4b, S4d). Hence, GST1-ARE-luciferase was used for further evaluation of PDT effect of CSNPs.

### Simultaneous measurement of ROS release and PDT effect of CSNPs *in-vitro*

**Figure 6:**
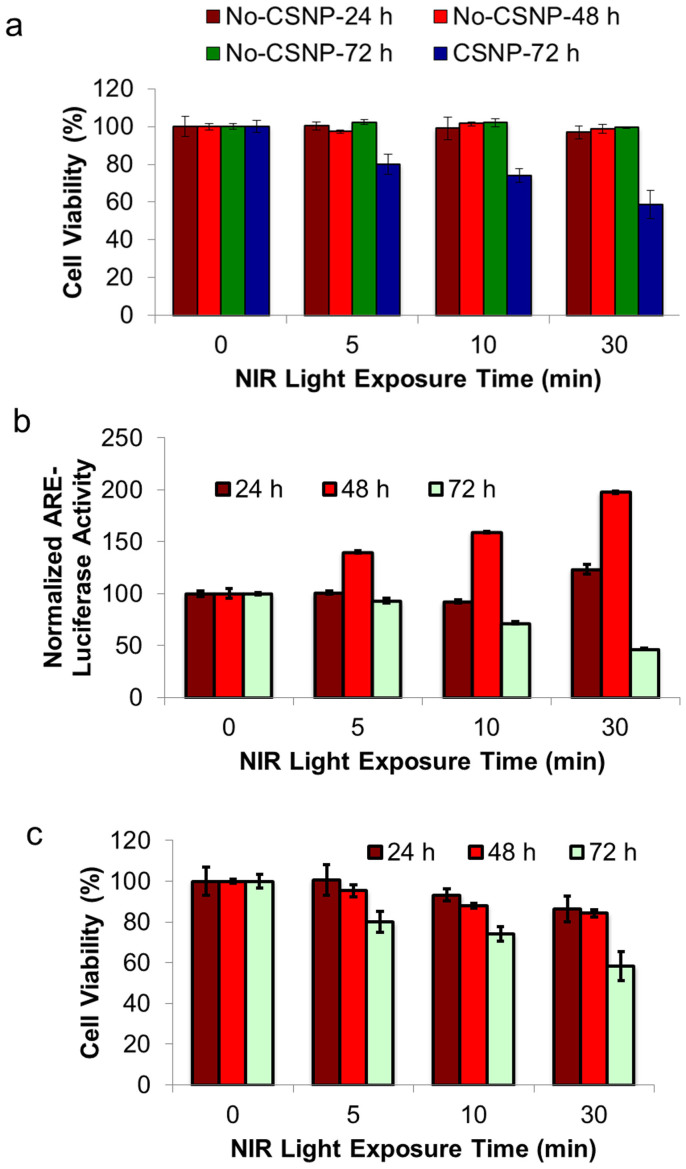
Effects of NIR light and CSNPs on cell viability. (a) NIR light toxicity to cells. Cell viability of MDA-MB-231cells after exposure to 980 nm NIR light (5 mW) in the presence and absence of 10 μg/mL of CSNPs. (b) ARE-Luciferase activity after different periods of NIR light exposure (0, 5, 10 and 30 min) and post-incubation times (24, 48 and 72 h). (c) PDT effect on MDA-MB-231cells. MTT assay of MDA-MB-231 cells incubated with 10 μg/mL of CSNPs for different periods of NIR light exposure (0, 5, 10 and 30 min) and subsequent post-incubation times (24, 48 and 72 h).

After the confirmation of little cytotoxicity of CSNPs and basal ROS level evaluation, the PDT effect of CSNPs in MDA-MB-231 breast cancer cells was studied upon 980 nm light (5 mW) irradiation at different times. Similarly, MDA-MB-231 cells treated with PBS and 980 nm NIR light exposure for the same period of time served as controls. Cell death is a complicated event, which is not just caused by ROS release. Therefore, instead of only using the post-treatment cell viability to evaluate the PDT efficiency, ROS release was simultaneously assessed by ARE-Luciferase assay. The MDA-MB-231 cells were transfected with GST1-Luciferase and subsequently incubated with 10 μg/mL of CSNPs. The transfected cells were exposed to 980 nm NIR light irradiation for different times (0, 5, 10 and 30 min). Then, the luciferase activity was performed in order to determine antioxidant signaling induced by ROS. Subsequently, the cell viability after NIR treatment was evaluated by MTT assay after 24, 48 and 72 h of incubation at 37°C, 5% CO_2_ in humidified atmosphere. The assay results (Fig. 6a) of control samples showed that the NIR light plays a rather minimal deleterious role in the breast cancer cells without the addition of CSNPs. In contrast, ~55% of cells remained alive for cells treated with 10 μg/mL CSNPs at 30 min NIR irradiation and 72 h post-irradiation incubation (Fig. 6a).

Figs. 6b, 6c show that 10 μg/mL of CSNPs without NIR irradiation did not affect cell viability and ARE-luciferase activity over time. Within 48 h post-irradiation incubation, there was a significant increase in luciferase activity, indicating increase of ROS release; cell viability dropped with respect to the irradiation time. It is interesting to note that although luciferase activity reached to a maximum at 48 h post-irradiation incubation time (Fig. 6b), the cells showed a maximum cell death at 72 h (Fig. 6c). This can be ascribed to the fact that the cells stopped further expression of ARE-luciferase protein due to cell death, and the initiation of apoptosis in cells can lead to cell death^33^,^34^. Thus, the remarkable reduction in the ARE-luciferase signal was observed in sample incubated for 72 h (Fig. 6b). The PDT effect (cancer cell killing efficiency of 45%) is clearly evident for CSNPs at the concentration of 10 μg/mL with 30 min NIR irradiation and 72 h post-irradiation incubation.

## Conclusion

We have developed a facile synthetic strategy for the fabrication of novel UCN@ZnO CSNPs for PDT. This design utilizes the emission of UCN for the sensitization of ROS generation with the aid of ZnO as photosensitizer, obviating the complicated procedures for the loading of photosensitizers. The unique core-shell combination of UCN and ZnO could be used for therapeutic application of deep-seated tumors. We have also shown the use of GST-Luciferase assay to measure ROS by monitoring antioxidant signaling of the cells, and MTT assay for *in-vitro* PDT effect on MDA-MB-231 breast cancer cells. The results of MTT assay showed that ~45–50% cell death (IC_50_) can be achieved for CSNPs at the concentration of 10 μg/mL with 30 min NIR irradiation and 72 h post-irradiation incubation, which may provide some indications in deciding the dosage concentrations required for *in vivo* PDT evaluation.

## Methods

### Synthesis of UCN@ZnO CSNPs

The CSNPs were synthesized in two steps including a UCN core (composed of NaYF_4_:Yb,Tm) synthesis and a ZnO shell formation. First, UCN was synthesized according to the literature methods^35^,^36^: YCl_3_ (0.8 mmol), YbCl_3_ (0.2 mmol), and TmCl_3_ (0.003 mmol) were mixed with 6 mL oleic acid and 15 ml octadecene (ODE) in a 100 mL flask. The solution was heated to 160°C to form a homogeneous solution, and then cooled to room temperature. A 10 ml methanol solution containing NaOH (2.5 mmol) and NH_4_F (4 mmol) was slowly added into the flask and stirred for 30 min. The solution was slowly heated to remove methanol, degassed at 100°C for 10 min, and then heated to 300°C and maintained for 1 h under Argon protection. After the solution was cooled naturally, nanocrystals were precipitated from the solution with ethanol and washed with ethanol/water (1:1 v/v) three times. Second, ZnO shell growth was carried out with the as-synthesized UCNs. Typically, 0.5 mmol of Zn(acac)_2_ and 1 mmol of 1,2-hexadecanediol were dissolved in 20 ml of diphenyl ether. 10 μl oleic acid and 100 μl oleylamine mixed with 0.05 mmol NaYF_4_:Yb,Tm in cyclohexane were then added into the solution and heated up to 100°C for 30 min. The temperature was gradually raised up to 160°C for 30 min and finally to 220°C for 1 h. After cooling to room temperature, the CSNPs were washed with acetone three times and collected by centrifugation at 8000 rpm.

### Water-soluble CSNPs via citrate ligand exchange

Sodium citrate at a concentration of 2 mmol was dissolved in 15 ml diethylene glycol. Fifty milligram of the CSNPs obtained from the previous step dispersed in 5 ml cyclohexane and 2 ml chloroform, was added into the sodium citrate solution and heated at 160°C for 3 h. After the solution was cooled down to room temperature, the CSNPs were treated with 0.1 M HCl solution and washed with acetone and water three times. The CSNPs were finally dispersed in water for further characterization and *in vitro* studies.

### Characterization of the CSNPs

TEM images were primarily obtained with a Philips EM300 electron microscope operated at an accelerating voltage of 300 kV. Elemental analysis was acquired with an energy-dispersive X-ray (EDX) equipped with the TEM. Scanning TEM (STEM) image was captured with an FEI Titan TEM with Schottky emitter operated at 200 kV. X-ray diffraction (XRD) patterns were collected by using a Bruker GADDS D8 Discover diffractometer with Cu Kα radiation (*λ* = 1.5418 Å). Fluorescence spectra were acquired with a SpectraPro 2150i fluorescence spectrometer equipped with a commercial 980 nm NIR laser. Room-temperature UV-visible absorption spectra were recorded with a Shimadzu UV-3150 UV/Vis spectrophotometer. Nano-zeta sizer measurements were performed on a Malvern zetasizer nano series.

### Reactive oxygen species (ROS) measurement

To 1 mg of citrated modified UCN@ZnO NPs dispersed in 1 mL ultra-pure water, 4 μL of 5 mM 3′-(p-aminophenyl) fluorescein (APF, from Invitrogen) was added and mixed in a vortex to introduce enough oxygen into the solution. After irradiation with 1.08 W 980 nm laser, 100 μL solution was centrifuged at 12000 rpm for 5 min. The supernatant (50 μL) was transferred into a 96-well black plate (Costar) to check the ROS by APF fluorescence evaluation. The fluorescence intensity of the APF dye was measured at 520 nm with 485 nm excitation using Fluostar Optima for different periods of time (0, 20, 40, and 60 min) upon continuous irradiation.

### Cell culture

Prior to experimentation, both 4T1 and MDA MB231 cell lines were separately grown under controlled culture conditions in 37°C incubators with 5% CO_2_. Each cell line was grown in sterile Corning® vented cell culture dishes with 8 mL of Gibco® Dulbecco's Modified Eagle Medium (DMEM) supplemented with 10% Fetal bovine serum, and 1% each penicillin and streptomycin. For experiments, we used 25,000 cells plated in a 24 well plate in 0.5 mL of complete medium/well.

### ARE-Luciferase transfections

We developed vector constructs express luciferase reporter gene under an antioxidant response element (GST1-Luciferase and NQO1-Luciferase). Following 24 h after plating, the cells in 24-well plates were transfected with ARE-Luciferase reporter constructs by using lipofectamine 2000 transfection reagent following manufacturers protocol (Invitrogen, USA). Renilla luciferase express under an ubiquitin promoter was co-transfected to normalize ARE-luciferase signal. We tested two different types of ARE-Luciferases (GST-Luciferase and NQO1-Luciferase) to best predict the downstream target gene of antioxidant signaling pathway for quick response to CSNPs induced ROS. In brief, to transfect 24-well plate the plasmids of GST-Luciferase or NQO1-Luciferase of 6.0 μg with 12 ng of Renilla Luciferase (R-Luc, for transfection normalization) were mixed with lipofectamine 2000 in 1:3 ratio in a serum free OptiMEM medium for complex formation. After 20 min, 50 μL (250 ng/well) of complex were added to each well. We changed the cells with a fresh medium after 24 h following transfection, and added CSNPs of required concentrations. After 30 min addition of CSNPs, the cells were exposed to 5 mW 980 nm NIR light to test CSNPs induced ROS release and its activation of ARE-Luciferase activity. Hotstuff 3 V 980 nm 5 mW Adjustable Focusable Infrared Laser Diode was used for cell studies.

### Luciferase assays

The 4T1 and MDA-MB-231 cells co-transfected with NQO1/GST-ARE-Luciferase and Renilla luciferase expressed under a constitutive ubiquitin promoter were used as luciferase assays after the completion of specific experimental conditions as in the following. The cells were lysed by adding 100 μL of 1×-passive lysis buffer (Promega, WI) by shaking for 15 min in a shaker. During this period, 1.2 mL of Luciferase Assay Reagent II (LAR II) was transferred into ice. The cell lysates transferred into 1.5 mL Eppendorf tubes were centrifuged at 10,000 g for 5 min at 25°C. After centrifugation, 20 μL of the supernatant from each tube were transferred into two fresh 1.5 mL micro-centrifuge tubes, one each for measuring Renilla and FLuc activities. Firefly and Renilla luciferase activities were measured by adding 100 μL of respective substrates (LARII for FLuc and Coelenterazine for Renilla luciferase) and measuring for 10 s in a Luminometer (Turner T20/20, Sunnyvale, CA). During this process, we also added 10 μL of supernatant from each sample into a 96-well plate, and measured protein concentration by using Bio-Rad protein assay reagent.

### MTT assay

Although the transfection experimentation for luciferase assay provides information on the amount of ROS released by the cells in response to CSNPs at various treatment conditions, it does not provide any information on cell death. Therefore, we performed MTT assay to measure cell proliferation/viability after various treatment conditions with CSNPs. The cells plated in 24 well plates after different treatment conditions for various time points were exposed to 200 μL of 5 mg/mL solution of 3-(4,5-dimethyl-2-thiazolyl)-2,5-diphenyl-2H-tetrazolium bromide (Sigma, St Louis) prepared in a phenol red free complete medium, and incubated further for 3 h at 37°C with 5% CO_2_. After incubation, the medium was aspirated carefully without disturbing newly formed precipitates, and 100 μL of dimethyl sulfoxide (DMSO) was added to each well. The plate was wrapped with aluminum foil and kept in the incubator for 15 min. The solutions in each well were transferred to separate 1.5 micro-centrifuge tubes, and centrifuged at 10,000 g for 5 min. Finally, 100 μL of solution was transferred into separate wells in a 96-well plate, and absorbance values were measured at 540 nm in a spectrophotometer.

## Author Contributions

S.T.S. and Y.Z. conceived the work and designed the experiments. Q.Q.D. performed the synthesis and measurements. A.R. and R.P. performed various reporter gene assays and toxicity studies in cell culture. All authors contributed to the analysis and interpretation of the experimental data. Q.Q.D., S.T.S. and R.P. wrote the manuscript with revisions from other co-authors.

## Supplementary Material

Supplementary Information Supplementary Information
