# Supplementary material for: Core – shell upconversion nanoparticle – semiconductor heterostructures for photodynamic therapy
Source: Sci Rep. 2015 Feb 5;5:8252. doi: 10.1038/srep08252 (PMC4317689; doi:10.1038/srep08252)
Supplement: Supplementary Information [file srep08252-s1.pdf]

Supplementary Information

Core – Shell Upconversion Nanoparticle – Semiconductor Heterostructures for Photodynamic Therapy

Qing Qing Dou[1], Adith Rangaramchandran[2], Subramanian Tamil Selvan[1,3], Ramasamy Paulmurugan[2] & Yong Zhang[3]

[1] Institute of Materials Research and Engineering (IMRE), A*STAR (Agency for Science, Technology and Research), 3 Research Link, Singapore 117602

[2] Molecular Imaging Program at Stanford, Bio-X Program, Stanford University School of Medicine, Palo Alto, California 94304, USA.

[3] National University of Singapore, Division of Biomedical Engineering, Faculty of Engineering, 7 Engineering Drive 1 Singapore 117576

Correspondence and requests for materials should be addressed to S.T.S. (email: subramaniant@imre.a-star.edu.sg) or R.P. (email: paulmur8@stanford.edu) or Y.Z. (email: biezy@nus.edu.sg)

The purity of UCN@ZnO CSNPs was justified from DLS measurements. The raw data obtained from nano-sizer for two different batches confirmed the purity of the UCN@ZnO CSNPs (See Figures S1, S2).

ZnO nanoparticles were also synthesized separately (without UCN) and the size was < 10 nm. In the CSNPs, if there are free ZnO NPs formed, there will be a peak around 10 nm in the size distribution curve. We did not see any peak around 10 nm. This confirms that our UCN@ZnO CSNPs are free from individual ZnO NPs.

			Diam. (nm)	% Intensity	Width (nm)
Z-Average (d.nm):	91.4	Peak 1:	107	100.0	45.3
PdI:	0.139	Peak 2:	0.00	0.0	0.00
Intercept:	0.966	Peak 3:	0.00	0.0	0.00

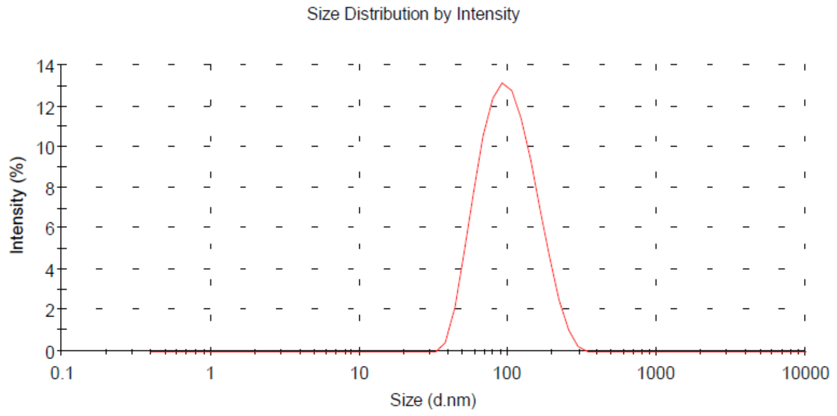

Figure S1. Size distribution of UCN@ZnO CSNPs confirming the purity from Batch 1.

			Diam. (nm)	% Intensity	Width (nm)
Z-Average (d.nm):	91.0	Peak 1:	103	97.3	44.6
PdI:	0.201	Peak 2:	4510	2.7	875
Intercept:	0.967	Peak 3:	0.00	0.0	0.00

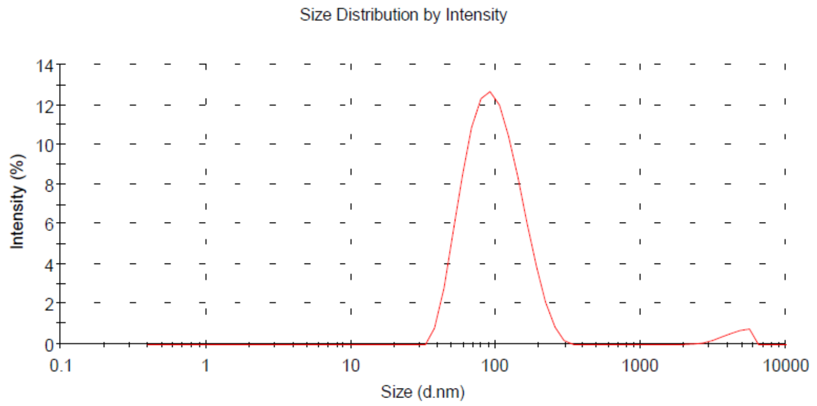

Figure S2. Size distribution of UCN@ZnO CSNPs confirming the purity from Batch 2.

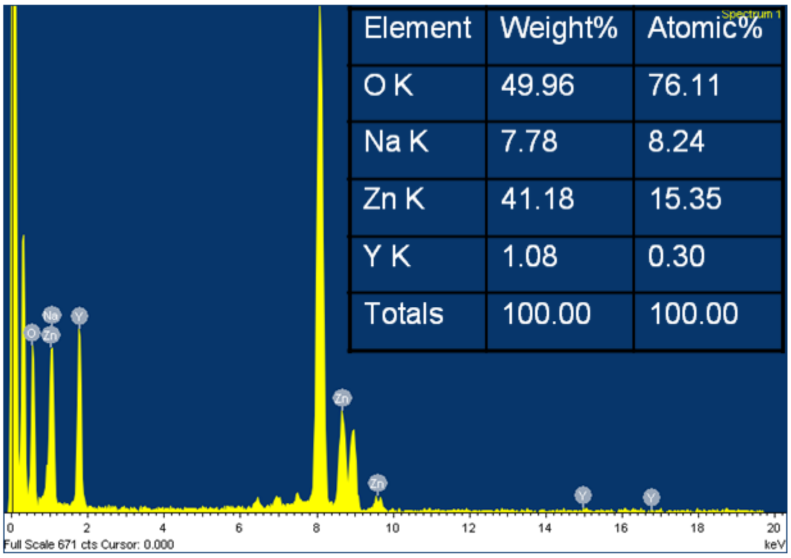

Element	Weight%	Atomic%
O K	49.96	76.11
Na K	7.78	8.24
Zn K	41.18	15.35
Y K	1.08	0.30
Totals	100.00	100.00

Figure S3. EDX spectrum of UCN@ZnO core-shell nanoparticles (CSNPs).

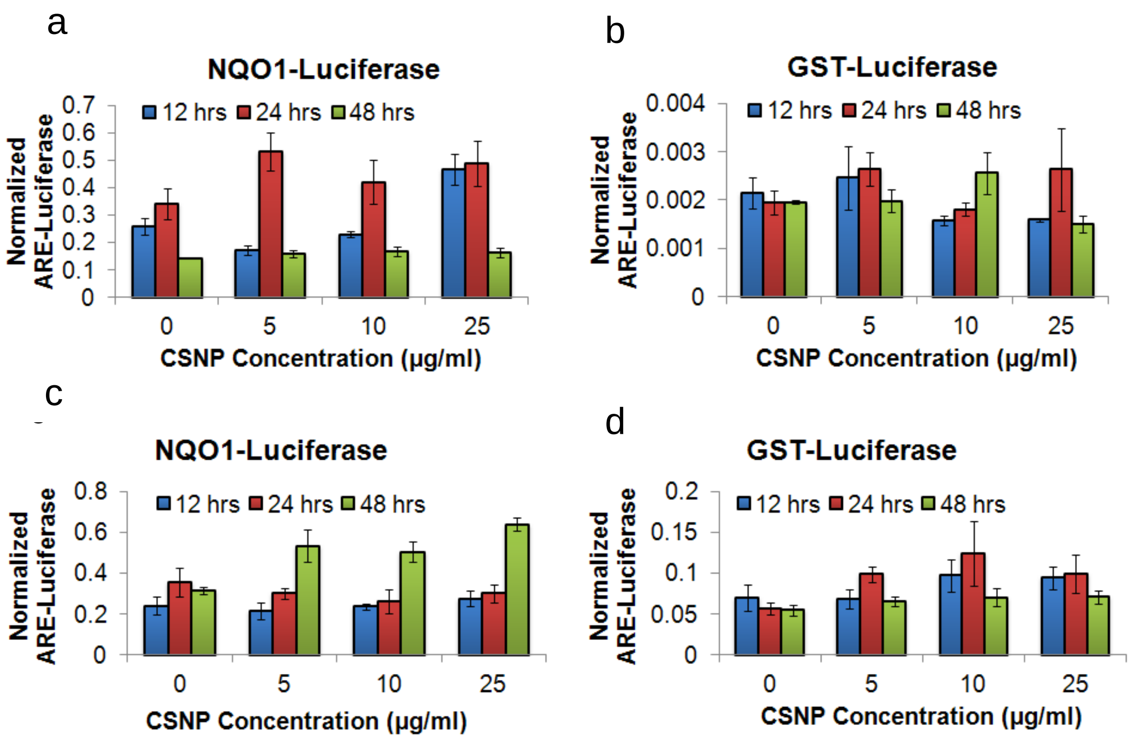

Figure S4. ARE-Luciferase level in response to the treatment of CSNPs in 4T1 (a,b) and MDA-MB-231 (c,d) cells before being activated by NIR 980 light. Each graph shows the luciferase readings and ROS activity measured at 12, 24, and 48 h after exposure to CSNPs. Both cell lines were exposed to CSNPs concentrations of 0, 5, 10, 25 μg/mL.
